# Predicted costs and benefits of eradicating BVDV from Ireland

**DOI:** 10.1186/2046-0481-65-12

**Published:** 2012-07-02

**Authors:** Alistair W Stott, Roger W Humphry, George J Gunn, Isabella Higgins, Thia Hennessy, Joe O’Flaherty, David A Graham

**Affiliations:** 1 Scottish Agricultural College, West Mains Road, Edinburgh, UK; 2 Centre for Veterinary Epidemiology and Risk Analysis, Veterinary Sciences Centre, University College Dublin, Dublin 4, Ireland; 3 Teagasc, Rural Economy Research Centre, Athenry, Galway, Co, UK; 4 Animal Health Ireland (AHI), Carrick-on-Shannon, Leitrim, Co, Ireland

**Keywords:** BVDV, Eradication, Cost benefit analysis

## Abstract

Bovine viral diarrhoea virus (BVDV) causes an economically important endemic disease (BVD) of cattle in Ireland and worldwide. Systematic eradication by detection and removal of infectious (BVDV carrier) cattle has been successful in several regions. We therefore assessed the benefits (disease losses avoided) and costs (testing and culling regime) of a potential eradication programme in Ireland. Published bio-economic models of BVDV spread in beef suckler herds and dairy herds were adapted to estimate potential benefits of eradication in Ireland. A simple model of BVDV spread in beef finisher herds was devised to estimate the benefits of eradication in this sector. A six year eradication programme consisting of 5 inter-related virological and serological testing programmes is outlined and costed. We found that the annualised benefits of BVDV eradication in Ireland exceeded the costs by a factor of 5 in the beef suckler sector and a factor of 14 in the dairy sector. Corresponding payback periods were 1.2 and 0.5 years respectively. These results highlight the significant economic impact of BVDV on the Irish cattle industry and suggest a clear economic benefit to eradication using the proposed approach. This type of cost-benefit analysis is considered an essential prerequisite prior to undertaking an eradication campaign of this magnitude.

## Background

Bovine viral diarrhoea virus (BVDV) causes BVD, one of the most important diseases of cattle worldwide [1]. This status results from a high prevalence in many countries [2] combined with wide ranging and insidious impacts on herd performance due to direct effects and to associations with infertility and with a range of other diseases through immunosuppression caused by BVDV [3]. The virus is spread primarily by individuals persistently infected (PI) with the virus. These animals become infected *in-utero* if their dam is either PI herself or susceptible to infection and exposed to the virus in early pregnancy, becoming transiently infected followed by seroconversion. Further details of the epidemiology and economics of BVD are given by Houe [2]. Vaccines are available but these add costs and farmers often fail to appreciate their limitations and the importance of correct and appropriate use [4]. There is also evidence that cattle farmers do not routinely apply the biosecurity practices necessary to prevent introduction of BVDV [5].

Given the above situation, systematic eradication of BVDV from a country or region offers an alternative approach to control at farm level that has been successfully applied in several European countries [6]. The most recently reported national BVDV eradication programme was described by Presi *et al.*[7]. They tested all Swiss cattle for BVD virus by antigen-capture ELISA or RT-PCR and culled all those individuals considered to be persistently infected (PI). Prevalence of virus-positive newborn calves fell from 1.8% to under 0.2% in two years. However, although the science and technology of BVDV eradication has been proven in Switzerland and elsewhere, the socioeconomic arguments are less well developed [8] but are likely to contribute greatly to a successful eradication campaign.

More *et al.*[9] set priorities for non-regulatory animal health in Ireland using Policy Delphi methods to elicit opinion from experts and farmers. They identified BVD as a disease that should be prioritized for action based on the current threat to animal health and the opportunity it presented for maximising the effective use of resources available to support animal health. Barrett *et al.*[10] subsequently reviewed the considerations for BVD eradication from the livestock industry in Ireland. They stressed the importance of cattle farming to the Irish economy, the threat which BVD poses to it and the potential for eradication provided that a systematic, aggressive and well coordinated programme is followed. This requires commitment from farmers, which in turn is dependent on good information provided by influential persuaders of the farming community such as veterinary surgeons, the farming press and farm advisers. This paper aims to provide an important part of this information i.e. a cost-benefit analysis (CBA) for a BVD eradication scheme for Ireland.

The specific objectives of this study were two-fold. The first was to estimate the benefits of freedom from BVDV to the Irish beef and dairy sectors at farm level. The annualised total current costs (losses) of BVD at farm level were taken as the benefits of eradication. The second objective was to estimate the costs of eradicating BVD from Ireland and thus complete the CBA.

## Methods

### Herd size and structure

Data processing was carried out using SAS® 9.1.3 (SAS Institute Inc., Cary, NC, USA). Numbers of herds and average herd sizes by enterprise type were estimated from the TB test returns collected in the year 2009. This method excluded those farms with no animals on the day of test and calves less than 6 weeks of age. Dealers, factory agents and feedlot herds were also excluded as they are not subject to TB test. Enterprise type was obtained from Animal Health Computer System test summary records.

### Model and data sources

To assess the benefits of BVDV eradication we used the bio-economic models described in Gunn *et al.*[11] and Gunn *et al.*[1] to predict the average total costs of BVD per cow per year in Irish suckler and dairy herds respectively. These papers were based on epidemiological and economic circumstances relevant to Scottish herds. We therefore adapted these models to Irish conditions by incorporating relevant Irish data. Unless otherwise attributed, economic data were taken from the Irish National Farm Survey (NFS) Data 2008 [12]. Although more recent data are available for 2009, they were not used here as 2009 was an exceptional year with output levels significantly lower than normal; at the time the current study was initiated, data for 2010 were not available. The NFS is a representative sample of Irish farms. In general, there are 1,100 farms in the survey each year which are weighted to represent the farming population of approx 110,000 farms. The NFS is collected as part of the Farm Accountancy Data Network of the EU. Other data were derived from the literature or databases cited, from personal communication attributed or otherwise from the expert knowledge of the authors themselves.

For beef finisher herds, not covered by the above models, we built a simple Markov chain model of the BVD virus flow necessary at herd level to sustain the reported prevalence of BVD in Ireland. Our model was built using spreadsheet software [13]. It allowed a costing of BVD impact to be estimated based on partial budgets adapted from the suckler herd. It also provided an estimate of the probability of BVD breakdown for use in the beef suckler model. For a description and explanation of Markov chain models in this context see Stott *et al.*[14].

### BVD in beef suckler herds

Latest updates of the model of Gunn *et al.*[11] described by Stott *et al.*[15] were incorporated. Main parameter settings for this exercise are shown in Table 1.

**Table 1:** Model parameters set to represent BVD in Irish suckler herds

**Parameter**	**Setting**	**Comment**
*Epidemiological parameters:*		
Prevalence of antibody positive herds	0.75	Estimated by AHI, range thought likely to be 0.65-0.85*.
Prevalence of herds with one or more PI	0.25	Estimated by AHI, range thought likely to be 0.15-0.35*.
Risk of virus entry to a farm in any one year	0.19	Based on beef finisher model (see text)
Proportion of heifers becoming transiently infected if naive herd infected.	0.75	0.75 in Gunn *et al.*[11]
Proportion of cows becoming transiently infected if naive herd infected.	0.75	0.75 in Gunn *et al.*[11]
*Financial parameters:*		
Sale value of normal male calf (€)	450	[12].
Sale value of normal female calf (€)	396	Relative to male weight in Gunn *et al.*[11]
Costs of immunosuppression effects (€/calf at risk)	3.06	Based on Gunn *et al.*[11]
Opportunity cost of farm labour (€/hour)	8.28	[12].
Replacement heifer cost (€)	1019	[12].
Value of cull cow (€)	793	[12].
Veterinary charges (€/hour or visit)	63	Estimate supplied by AHI
Other minor assumptions converted from £ to €	1.1322	Currency conversion factor

*Figures were derived primarily from unpublished data provided from a number of sources.

This model draws herds of fixed size at random from a population of herds set up to represent the epidemiological situation of beef suckler herds in the country or region concerned (for further details of this see [16]). This means that some herds are naïve, others contain one or more PIs and the rest have variable proportions of Ab + and susceptible individuals. The model then tracks each herd forward in time for 10 annual steps. At each step, animals enter and leave the herd, and calves are born. The model adjusts heifer retention rates each year to ensure that herd size remains constant. If the herd contained a PI at the start of the simulation then susceptible individuals may become transiently infected. Some of these cases may be pregnant heifers or cows, which may give birth to PI calves. It is also possible for PIs to die prematurely or be sold so that no virus is then circulating on the farm. However, it is also possible for BVDV to arrive spontaneously as a consequence of biosecurity breakdown. The financial consequences of these events such as the lost performance of transiently infected calves, infertility of cows, premature culling, immunosuppression of calves, extra farm labour and veterinary costs etc. are accumulated over the 10 year period and then annualised (their net present value is expressed as an annuity based on the discount rate of 0.05) and expressed per cow for easier interpretation. As the outcome depends on a series of chance events, each iteration of the (stochastic) model provided a different cost of BVD. The model was therefore run many times (500 in this study) for a range of representative herd sizes to build up a national picture of the average farm level disease costs.

Available data [17] suggests that 9.3% of suckler herds in Ireland used BVD vaccine in 2010. To take this into account, we repeated our model runs for vaccinated herds. We assumed that vaccine costs €3/dose with cows needing 1 dose and heifers 2 doses. The vaccine was assumed to be 90% effective i.e. 0.9 of BVD susceptible cows and heifers would become immune by its use (based on [18]). Using outputs from model runs both with and without vaccination, we were able to adjust our estimates of the national losses due to BVD downwards to account for current vaccine usage.

### BVD in dairy herds

Further details and updates to the model of [1] are given by [19]. Main parameter settings for this exercise are shown in Table 2. The model works in similar fashion to the beef suckler model but with a shorter (quarterly) time step. These shorter time steps reflect the seasonal nature of milk production in contrast to the annual cycle of suckled calf production. Unlike the beef suckler model, the starting epidemiological scenario is fixed, with the model runs being performed twice, once assuming that the herd contains a PI cow at the start of the simulation, the other assuming that it is naïve. As before, BVD virus entry is still possible later on in the simulation due to purchase of PI replacements and/or other forms of biosecurity breakdown. By comparing the losses due to BVD under these two extreme starting scenarios, an overall impression of the financial impact of the disease in the dairy sector can be obtained. Vaccination was not an option in the dairy herd model. Estimates of the impact of vaccination on losses due to BVD in this sector were therefore extrapolated from the suckler herd results based on an approximate 40% uptake at herd level [17].

**Table 2:** Model parameters set to represent BVD in Irish dairy herds

**Parameter**	**Setting**	**Comment**
Prevalence of PI in purchased animals	0.0075	Supplied by AHI (Table 1)
Probability of biosecurity breakdown to BVD in any one year	0.133	Based on Graham *et al.*[20].
Milk price (€/litre)	0.294	CSO Ireland. Average 3.7% BF milk price for 2010.
http://www.cso.ie/px/pxeirestat/Statire/SelectVarVal/Define.asp?maintable=AJM06		
Replacement heifer cost (€)	1,070	[12].
Value of cull cow (€)	799	[12].
Veterinary & Medicine charges (€/cow/year)	64	Estimate supplied by AHI

### BVD in beef finisher herds

Given the nature of the disease, BVD virus will emerge from PI calves born to cows in breeding herds. However, some of these calves will be sold to store rearing/finishing herds where such animals will cause financial loss through their own morbidity/mortality and through spread of the virus to susceptible herd mates. It was therefore important to estimate the additional financial losses to BVD arising from these herds. A simplified version of the models described above was therefore developed based on the same Markov chain principles applied in them. Unlike the two breeding herd models, which use Markov chains to follow the transitions of cattle between disease states (susceptible, PI, transiently infected and immune) within a herd, this model follows the transition of herds between 4 states based on how recently they were exposed to BVD virus and therefore on how many susceptible animals they contain. In another contrast to the other two models, this one was first operated in reverse i.e. starting with the expected proportion of herds in some states as explained below; the model determined the disease transmission probabilities required to achieve this outcome. The main output from this Markov chain model was therefore its transition matrix as well as the final state vector. The field based assumptions on which this model was based and the transition probabilities emerging from it were used in the other two models, ensuring consistency between them and allowing their epidemiological assumptions to be matched with best estimates of the epidemiological situation observed in the field. Once the transition matrix was determined, the model could be run to predict the proportion of herds in each state. The number of animals likely to be affected by BVD in various ways could then be found and the financial losses arising from them could be calculated.

Based on information supplied by AHI (D Graham, unpublished data) for beef suckler herds (Table 1) it was assumed that 0.75 of beef finisher herds would be antibody positive for BVDV with 0.25 of these containing a PI animal (active infection and hence financial loss). It was thought that in practice, very few beef or dairy herds in Ireland would be entirely naïve i.e. contain no animals that were Ab + and therefore immune due to exposure at some point in the past to BVD virus. However, preliminary modelling work indicated that the cost of virus entry into a herd with few (less than 0.25) Ab + animals would be little less than a totally naïve herd. We therefore conceived of 4 basic finisher herd types in terms of their extent of Ab + and PI animals. In addition to the 0.25 of PI herds, other herds without a PI might be recently exposed to virus (Ab + Recent) with about 0.75 of all animals Ab+. Other Ab + herds may have been exposed less recently (Ab + Older) and thus contain 0.25 animals Ab+. The 0.25 of remaining herds i.e. with less than 0.25 of animals Ab + would be designated naïve.

The transition matrix for the Markov chain model which delivers 0.25 of herds PI and 0.25 of herds naïve is shown in Table 3. The spreadsheet’s inbuilt optimisation algorithm (solver) was used to obtain these parameters and hence derive the transition matrix. The probability of all herds suffering a new virus entry each year i.e. becoming PI if not already PI was found to be 0.19. The model also predicted that over the year, 0.32 herds would be Ab + Recent and 0.18 Ab + Older. The full breakdown of our 4 herd types was therefore 0.25, 0.32, 0.18 and 0.25 for PI, Ab + Recent, Ab + Older and naïve respectively. On this basis we were able to estimate the overall annual cost of BVD in this sector based on previously estimated losses from PIs (€594) and transiently infected animals (€166). These estimates were based on the assumed impacts of BVD on suckled calves given in Gunn *et al.*[11] as updated by Stott *et al.*[15] but applied to finishers. The healthy finisher was assumed to weigh 600 kg (male) or 500 kg (female), valued at €1.53/kg and €1.34/kg respectively (obtained from CSO Ireland, [17] prices). The healthy finishers were assumed purchased as stores weighing 250 kg and 220 kg for male and female respectively, growing at 0.93 kg and 0.86 kg per day. Transiently infected cattle (TI) were assumed to lose 10% of these prices and growth rates, while PIs would lose 20% (based on estimates derived from expert panels consulted during the construction of the model described by [11]). In addition, TIs would attract extra vet bills of €11 and require additional farm labour of 1.1 hours (priced as shown in Table 1). The equivalent figures for a PI were €110 and 10 hours respectively.

**Table 3:** Transition matrix for the Markov chain model of beef finisher herds*

	**PI**	**AB + Recent**	**AB + Older**	**Naïve**	**Total**
PI	0.43	0.57	0.00	0.00	1.0
AB + Recent	0.19	0.55	0.26	0.00	1.0
AB + Older	0.19	0.00	0.55	0.26	1.0
Naïve	0.19	0.00	0.00	0.81	1.0

* Rows represent herd types at time t and columns herd types at time t + 1. For an explanation of herd types see text.

Note that the data used to establish the number of herds affected and the number of cattle at risk in beef finisher herds were obtained from a breakdown of data by herd size. The size categories were 1–20, 21–50, 51–100, 101–250, 251–500 and >500. Cattle numbers were assumed to be the mid-point in each category except for the largest herd size category, which was assumed to be 500 head i.e. a conservative estimate. Herds categorized as ‘beef’ and as ‘other’ were assumed to be beef finisher herds.

### Model validation and sensitivity analysis

Validation of the two breeding herd models is described in the source papers cited earlier [1,11]. In both cases, model results were compared with cross sectional field data of BVD outbreaks available at the time and were also subject to iterative expert evaluation. Since then, longitudinal data following the progress of BVD on commercial farms in financial as well as epidemiological terms are becoming available (Ganser *et al.,*[21]). These confirm the general extent and variability of the financial impacts of BVD reflected in our model results. Furthermore, by linking our breeding herd models to field observation via our finisher herd model as described above, we were able to establish a more coherent set of results than would otherwise be possible, linked to the epidemiological situation observed in the field.

To investigate the influence of the value assigned to each of the breeding herd model parameters, a sensitivity analysis was carried out by re-running the model with each value individually increased by 10%. This identified the parameters with the greatest potential influence on results. This information when combined with the expected variation in parameter estimates provided an indication of how robust the BVD loss estimates were likely to be. As a further guide to this, the breeding herd model outputs at the lowest 10^th^ percentile (i.e. 90% of runs gave a higher loss than this) were used to estimate the minimum (‘best case’) BVD losses.

### Costs of eradicating BVD from Ireland

For the purposes of costing, a six-year programme for BVD eradication from Ireland was broken down into five test regimes with associated costs as follows, each applied to beef suckler and dairy herds. Any PIs in beef finisher herds were assumed to clear through slaughter and natural wastage.

Test 1 (Tag). This refers to the testing of ear tissue samples collected from all calves by tissue test tags as part of the official identification process and tested for BVD virus (antigen or RNA). This was considered to be carried out annually for the first three years of the programme. Each test was estimated to cost €4 plus a further €0.6 for the tag (an additional cost relative to the cost of the conventional official identification tag). Given that a PI cow will inevitably produce a PI calf, the dam of any calf returning a negative virus result can therefore also be considered to be non-PI. After one round of negative testing, the calves and the majority of the adult cattle (excluding those that did not produce a calf, or that are male/bulls), can be considered to be non-PI. The first round of testing will not give a clear indication of the status of any yearlings in the herd (assumed to be primarily female breeding stock). After a second round of negative testing the following year, that part of the adult herd that has produced a calf that year is again also considered to not contain PIs. This year the yearling stock from year 1 will also have entered the breeding herd, giving a result for these animals by trace back from their offspring. The pool of animals without any BVDV status is expected to shrink or disappear. After a third clear round of testing, this group with no BVDV status (based on direct testing or indirectly from their calves’ results) is expected to disappear in the majority of herds. Where such animals exist, a ‘completion test’ (assumed for this exercise to be based on blood samples collected by the herd’s veterinary surgeon) will be carried out (Test 2 below). Successful implementation of this approach is based on the assumption that parentage of calves is correctly assigned.

Test 2 (Completion). This test will be applied to herds with animals of unknown status after completion of the test 1 phase. These animals will be identifiable for each herd using the ICBF database. Based on analysis of current herd data, it was assumed that after 3 years, 0.44 of dairy herds and 0.33 of suckler herds will require a completion test (Sean Coughlan, personal communication). It was further assumed that on average three animals in each of these herds will require extra blood tests at €4 each plus an associated veterinary visit at €63. The small number of animals per herd assumed to require this test reflects the influence of the on-going testing programme, which will limit the numbers of animals of unknown status remaining at this stage of the programme.

Test 3 (PI removal). This test will be applied to herds returning one or more virus positive calves under test 1. It was assumed that 0.25 of herds (Table 1) have on average 1.7 PI’s. This is the approximate number of PI’s per herd needed to reach an animal level prevalence of 0.0075 (Table 1) if herd prevalence is 0.25. For simplicity this process was assumed to occur in year 1 only, although in practice the process would take longer but tail off towards the end of the eradication programme. All animals would be virus tested by blood sample in these herds at the cost of €4 per test plus associated veterinary visit cost of €63. Based on Presi *et al.*[7], 0.8 of PIs detected would be calves. In the dairy herd all PI calves were assumed to be disposed of at an average cost of €60. For the adult PIs in the dairy herd, 0.9 were assumed to be fit for slaughter at the normal cull value, giving a net replacement cost of €271 (Table 2). The remainder were assumed to attract a disposal charge of €150. This resulted in an expected (probability weighted average) cost of PI disposal in the dairy herd of €121/head. This figure is made up of 0.8 calf disposals at -€60 = −€48 plus 0.18 (0.2x0.9) adults replaced fat at a net cost of -€271 = −€48.78 and finally 0.02 (0.2x0.1) adults replaced after disposal at a net cost of -€1220 = −€24.40.

A similar process was used for PI disposal cost in suckler herds based on the same assumptions as for dairy but with cow replacement price modifications as in Table 1 (net replacement cost of a suckler cow is €226). Using this approach, the expected disposal cost would be €112/head (0.8x-€60 + 0.18x-€226 + 0.02x-€1169). Note that the costs and benefits of purchasing a replacement foster calf were not included. For both dairy and suckler herds it was assumed that virus positive cattle would be removed within weeks of their being identified.

Test 4 (Surveillance). These costs cover the costs of monitoring the national herds for re-entry of BVDV following successful completion of the test 1 regime. Each year for years 4 to 6 of the programme, every dairy herd was assumed to require three bulk tank milk (or first lactation screen) BVD antibody tests at three to four-monthly intervals costing €6/test. An equivalent blood test was assumed to be carried out on 10 animals in every beef suckler herd, at a cost of €4/test. No additional veterinary related charges were included for the suckler herds as this surveillance blood test could be carried out at the TB test visit. However, a cost of €30 to cover reagents, shipment etc. was assumed.

Test 5 (Confirm PI): It was assumed that in herds where PIs were identified (Test 3), 0.7 of these PIs would be subjected to a confirmatory virus test costing €4 with an associated veterinary fee of €63.

The costs of all 5 tests were summed across six years for dairy and beef sucklers and expressed in net present value terms (NPV) using an assumed real interest rate of 0.05. The NPV was then taken as the estimated cost of eradication. As the details of eradication are uncertain and subject to change, costs are broken down into their component parts in the results tables for each test so that variations can be easily computed.

## Results

### BVD impact in beef suckler herds

The estimated average annual output losses due to BVD in the Irish suckler herd are shown in Table 4. The average costs per cow per year in herds of different sizes are aggregated in Table 4 to give a figure for the national herd before accounting for the net benefit of vaccine use. The average costs per cow per year in small herds (<51 animals) was higher at €38/cow/year than in larger herds at €29/cow/year. When the model was re-run assuming use of vaccine, losses due to BVDV per cow per year including the cost of vaccine fell to €14/cow/year in smaller herds and €10-11/cow/year in larger herds. Vaccination costs incorporated in these figures were €4.80/cow/year. This equated to a net national saving of €1.7 m/year due to the use of vaccines based on current usage rates. The overall costs of €29 m/year shown in Table 4 can therefore be reduced to €27 m/year.

**Table 4:** Estimated current average annual losses from BVD in Irish dairy and suckler herds*

**Herd type**	**Mean cows/herd**	**Number of herds**	**Number of cows**	**Costs/cow/yr (€)**	**Total costs (€m)**
Suckler	14.2	63,770	905,110	32	29.1
Dairy**	47.0	24,267	1,140,533	63	71.7

* Before accounting for the net benefit of vaccine use.

**Average of naïve herd costs at €57/cow/year and PI herd costs at €69/cow/year.

The sensitivity of the results given in Table 4 to changes in model parameters given in Table 1 are shown in Table 5. The sensitivity analysis was based on a beef suckler herd of average size (14 cows). The average output loss from BVD in this herd was €30.49/cow/year. The most sensitive assumptions are those relating to risk of virus entry, prevalence of herds with one or more PIs and the cost of a replacement heifer. The ‘best case’ (10^th^ percentile) output loss from this model averaged €3.18/cow/year over the various herd size assumptions. This gave a total annual cost for the sector of approximately €3 m.

**Table 5:** Sensitivity of suckler herd results in Table 4 to 10% increase in the estimating model’s parameter settings

**Parameter**	New setting*	**Percent change in costs/cow/year**
*Epidemiological parameters:*		
Prevalence of antibody positive herds	0.825	0.6
Prevalence of herds with one or more PI	0.275	7.4
Risk of virus entry into a herd in any one year	0.209	7.8
Proportion of heifers that become transient infected if naïve herd infected	0.825	1.3
Proportion of cows that become transient infected if naïve herd infected	0.825	5.1
*Financial parameters:*		
Sale value of normal male calf (€)	495	5.5
Sale value of normal female calf (€)	436	5.2
Costs of immunosuppression effects (€/calf at risk)	3.4	1.4
Opportunity cost of farm labour (€/hour)	9.11	5.6
Replacement heifer cost (€)	1,121	7.5
Value of cull cow (€)	872	−1.0
Veterinary charges (€/hour or visit)	69	2.8
Other minor cost assumptions converted from £ to €	1.24	0.0

*See Table 1 for original parameter settings.

### BVD impact in dairy herds

Table 4 also shows the estimated average annual output losses due to BVD in the Irish dairy herd, assuming that herds are either all naïve at the start of the simulated epidemic or already contain a PI cow (infected herd). As approximately equal proportions (0.25, 0.25) of herds are thought to be naïve or contain a PI, we took the mean of these 2 estimates (€72 m) as the approximate average annual losses due to BVD in the Irish dairy industry i.e. about €63/cow. Vaccine use is considered to be about four times greater in Irish dairy herds (assumed 40% of herds) than it is in Irish suckler herds. A proportionately greater saving (24%) due to vaccine use may therefore be expected in the dairy sector compared to the beef suckler sector. Allowing for vaccination use then reduces the national annual output loss due to BVD to €55 m i.e. €48/cow.

Sensitivity analysis for the dairy sector results appear in Table 6. This analysis shows that results are most sensitive to milk price and biosecurity breakdown in naïve herds. The ‘best case’ (10^th^ percentile) output loss from this model averaged €52/cow/year over the various herd size and starting scenario assumptions. This gave a total annual cost for the sector of approximately €60 m (€46 m after allowing for vaccine use).

**Table 6:** Sensitivity of dairy herd results in Table 4 to 10% increase in the estimating model’s parameter settings

**Parameter**	New setting*	**Naïve herd**	**Percent change in costs /cow/year**
Prevalence of PI in purchased animals	0.00825	1.0	0.5
Probability of biosecurity breakdown to BVD in any one year	0.1463	4.3	1.7
Milk price (€/litre)	0.323	6.5	6.4
Replacement heifer cost (€)	1,177	2.1	1.0
Value of cull cow (€)	879	−0.1	−0.3
Veterinary & Medicine charges (€/cow)	70	−1.5	0.0

*See Table 2 for original parameter settings.

### BVD impact in beef finisher herds

A summary of the impacts of BVD across this sector in Ireland are given in Table 7. The majority of the losses accrue perhaps surprisingly to herds that have had no recent exposure to the virus (AB + Older and Naïve). This is because although the risks of exposure in any one year are not high (0.19) there are a lot of herds in this category (0.43) and the high numbers of susceptible animals in these herds make the losses per head greater should infection be introduced. In beef finisher herds containing a PI, other animals are assumed to have already acquired immunity and the apparently low costs per animal are a reflection of this. The final category (recently exposed AB + Recent) have relatively few animals that are not immune and therefore if re-exposed to the virus, average losses per head are lower. The total number of animals in this sector is approximately 1.06 m, giving an overall annual loss from BVD of 19 €/head in this sector.

**Table 7:** Estimated current average annual BVD losses in Irish beef finisher herds

Herd type*	**Number of herds affected**	**Cattle at risk (head)**	**Costs/head/yr (€)**	**Total costs (€m)**
PI	6,387	265,078	18	4.7
AB + Recent	1,532	107,218	25	2.7
AB + Older	885	36,712	125	4.6
Naive	1,208	50,150	166	8.4
Total:	10,012	459,158		20.4

*PI-Herds with a PI, AB + antibody positive. For further description of herd types see text.

### Costs of BVD eradication

A breakdown of the cost calculations for each of the 5 tests needed to establish freedom from BVDV in Irish suckler herds are shown in Table 8. A similar breakdown for dairy herds is shown in Table 9. These results form the basis for the NPV calculations which appear in Tables 10 and 11 for suckler herds and dairy herds respectively. The annuity equivalents of the NPVs were approximately €6 m and €5 m for suckler and dairy herds respectively. Overall the total cost of eradication over the 6 year period was €55 m (€32 m suckler, €23 m dairy).

**Table 8:** Predicted costs/year for each test required during BVDV eradication from Irish suckler herds

T*	**Number of herds**	**Tests/herd**	**Tests**	**Cost/Test (€)**	**Vet Fee (€)**	**Culls**	**Costs/cull (€)**	**Other Fee (€)**	**Total Costs (€m)**				
									**Tests**	**Vet**	**Replace PIs**	**Other fees**	**Total**
1	63,770	14.4	918,288	4.6					4.22				4.22
2	21,044	3.0	63,132	4.0	63				0.25	1.33			1.58
3	15,943	50.1	798,719	4.0	63	27,102	112		3.20	1.00	3.04		7.24
4	63,770	10.0	637,700	4.0				30	2.55			1.91	4.46
5	15,943	1.2	18,972	4.0	63				0.08	1.20			1.28

*Test 1: Tag calves, 2: Herd completion, 3: Remove PIs, 4: Surveillance, 5: Confirm PIs.

**Table 9:** Predicted costs/year for each test required during BVDV eradication from Irish dairy herds

T*	**Number of herds**	**Tests/herd**	**Tests**	**Cost/Test (€)**	**Vet Fee (€)**	**Culls**	**Costs/cull (€)**	**Other Fee (€)**	**Total Costs (€m)**				
									**Tests**	**Vet**	**Replace PIs**	**Other fees**	**Total**
1	24,267	47.0	1,140,549	4.6					5.25				5.25
2	10,677	3.0	32,032	4.0	63				0.13	0.67			0.80
3	6,067	142.0	861,479	4.0	63	10,313	121.18		3.45	0.38	1.25		5.08
4	24,267	9.0	218,403	6.0					1.31				1.31
5	6,067	1.2	7,219	4.0	63				0.03	0.45			0.48

*Tests as for Table 8.

**Table 10:** Net present value (NPV) cashflows for proposed BVD eradication programme in Irish suckler herds (€m)

**Tests:**						
**Year**	**1**	**2**	**3**	**4**	**5**	Total*
	**Tag calves**	**Herd completion**	**Remove PI**	**Surveillance**	**Confirm PI**	
1	4.22		7.24		1.28	12.74
2	4.22					4.22
3	4.22	1.58				5.80
4				4.46		4.46
5				4.46		4.46
6				4.46		4.46
NPV:						31.47

*Row totals are before discounting and therefore their sum exceeds the NPV.

**Table 11:** Net present value of proposed BVD eradication programme in Irish dairy herds (€m)

**Tests:**						
**Year**	**1**	**2**	**3**	**4**	**5**	Total*
	**Tag calves**	**Herd completion**	**Remove PI**	**Surveillance**	**Confirm PI**	
1	5.25		5.08		0.48	10.81
2	5.25					5.25
3	5.25	0.80				6.05
4				1.31		1.31
5				1.31		1.31
6				1.31		1.31
NPV:						23.36

*Row totals are before discounting and therefore their sum exceeds the NPV.

### CBA of BVDV eradication from Ireland

Table 12 compares the costs of eradication with the benefits of BVD losses saved first by comparing annual benefits with annualised costs (annuity equivalent of NPV) and then as a pay-back period over the six year period of the proposed programme. The annual benefits of eradication exceed the annual costs by a factor of 5 in the suckler herds and by a factor of 14 in dairy herds. The pay-back period is the NPV of eradication costs divided by the annualised BVD losses i.e. the length of time it will take to recoup the total (6-year) costs of eradication from the (annual) BVD losses. The eradication costs in the suckler herd over a six year programme are approximately equal to the losses that occur each year. In the dairy sector, the benefit is even greater, with the costs of the 6 year eradication programme equalling less than 40% of the losses that occur each year. In the absence of a programme, these losses can be assumed to continue year on year for the foreseeable future. Even if the ‘best case’ assumptions are made about the breeding herd BVD losses and any losses in finisher herds are assumed negligible, eradication costs are still covered by the benefits. In this case the total benefits (BVD losses saved) are €49 m (€3 m from suckler herds plus €46 m from dairy herds). This gives a cost benefit ratio of almost 5 and payback of total eradication costs of just over 1 year.

**Table 12:** CBA and payback period for eradication of BVDV from Ireland

	**Suckler**	**Dairy**	**Sector:Beef**	**Total**
Benefit (BVD losses saved*) (€m/year)	27	55	20	102
Cost (test costs) (€m/year)	6	4		10
Benefit cost ratio	5	14		10
Total eradication costs (NPV €m)	32	23		55
Payback period (NPV/annual benefit) in years	1.2	0.4		0.5

*includes adjustment for estimated savings through use of vaccination.

## Discussion

Our results predict that benefits of BVDV eradication from Ireland using the proposed approach far exceed the costs. This result occurred despite building in various conservative assumptions about the cost of eradication. First, the completion test (test 2) may be carried out as part of routine herd visits or at TB test, thus reducing or removing the veterinarian’s visiting charge. The number of herds requiring a completion test is probably overestimated as it was based on current figures rather than the reduced number once more animals moving have a tag test result confirming them as non-PI. Test 3 (PI removal) and test 5 (confirm PI) may also be less costly as cheaper alternatives to the blood testing protocol may be incorporated in the programme. Surveillance test costs (test 4) may also be reduced by pooling samples. Various other costs may be reduced by the economies of scale associated with a nationwide eradication programme. On the other hand, we only included direct financial costs in our assessment of the costs of eradication. There will be other costs that are less easy to assess but could increase costs substantially. For example, premature culling implies loss of future profits that are not captured in the net cash cost of replacement. Such costs are often included as retention pay-off (RPO), which estimates the difference in profit between replacement at the optimal time and immediate replacement [22]. However, in the case of the immediate replacement of a PI, the RPO will probably be negative due to the future disease costs avoided. We have accounted for this on the benefits (BVD losses avoided) side. Any outstanding costs not captured by our direct approach (e.g. opportunity costs of farm labour, scheme administration costs etc.) are likely to be outweighed by uncaptured benefits of eradication such as improved animal welfare, better biosecurity, enhanced reputation of the farming sector and the experiential knowledge gained by those involved [23].

It is possible that our estimates of the BVD losses saved (benefits) may not be fully realised in practice. However, even if we took the losses at the 10^th^ percentile of our range of loss estimates rather than the mean, the cost benefit ratio and payback periods were still attractive. Furthermore, based on our sensitivity analysis, substantially lower BVD losses saved would arise either from lower prevalence of PIs/risk of virus entry and/or from lower commodity prices. In the first case, lower BVD prevalence will reduce eradication costs thus partially offsetting the impact of reduced losses avoided. In the second case, commodity prices have risen since the price assumptions used here were taken. Nevertheless, it will always be difficult to assess *ex-ante* the benefits of an eradication programme. In other countries where BVD eradication has been implemented, *ex-post* assessment of progress has been largely in epidemiological rather than economic terms (e.g. [7]). A notable exception is Norway [23]. In this case, benefits (measured as the difference between observed and expected BVD losses had no eradication scheme been in place) exceeded costs in all years reported (1993 to 2002). The cost benefit ratio of just over 1 in 1993, peaked at 12 in 2000. These results are comparable with our own. The Scottish Government have provided an *ex-ante* financial assessment of their BVD eradication programme [24,25]). However, their results are less comparable with those reported here since they are based on assumed impacts on farm business performance rather than on the cost and benefits of BVD eradication *per se*. Even so, the Scottish Government report a positive economic assessment and their eradication programme is currently underway.

Despite our positive outcome in favour of BVD eradication from Ireland, investment appraisal is hampered by the different basis of the cost and benefit calculations. Costs of eradication are finite, can be fairly accurately estimated and must be incurred by all concerned in advance and without guarantee of success. By contrast, the benefits of eradication are based on uncertain and often hidden losses from BVD that will vary considerably between farms but persist indefinitely in the absence of control. There is the added difficulty that the incentives to eradicate will vary as an eradication scheme progresses and may mitigate against progress at the start when risks of re-infection are high and at the end when the marginal cost of eradication increase with the proportion of difficult cases remaining [1]. Furthermore, the attitudes of farmers towards BVD eradication vary widely between country, region and locality and are an important factor in the success of eradication campaigns [8]. It follows that experience of other countries and CBA are just parts of the process needed to implement a successful eradication campaign. However, a high benefit-cost ratio is a highly desirable prerequisite. This assessment of the components of this ratio is therefore important.

The estimated output losses due to BVD in Ireland provided here are expected results i.e. the average of a large number of model runs and yet we do not provide any estimates of uncertainty. The models used do capture some of the uncertainty associated with the probabilistic processes of the disease (e.g. uncertainty of a PI being born to a transiently infected cow). They do not however take into account the uncertainty associated with any other factors (e.g. financial assumptions). Therefore it is inappropriate to provide summaries of uncertainty since they would be insufficiently conservative (i.e. too small) and imply a higher degree of confidence than is warranted. Instead we have provided a sensitivity analysis for each of the main assumptions in our models. Our results are only sensitive to one or two key epidemiological and financial assumptions such as the probability of virus entry (biosecurity breakdown), milk prices and replacement heifer costs. However, as benefits greatly exceed costs in this exercise these are not as critical as they might have been if costs and benefits had been more closely matched.

It is important to consider that the eradication programme may not be successful or BVD may re-enter the country after eradication leading to renewed losses. To prevent this latter occurrence, greater expenditure may be required on biosecurity precautions. This may increase the costs of eradication above those recorded here. However, it may also give collateral benefits. The productivity gains that accompany BVD eradication may depress market prices and so partially offset the benefits to farmers of eradication as shown by [19] for Scotland. This may be less of a problem for a predominantly food exporting country like Ireland, especially if BVD eradication gives a competitive advantage in overseas markets where BVD persists. The productivity gains are likely to reduce the environmental impact of agriculture through reduced emissions of greenhouse gases per unit of product [26]. Freedom from BVD will also bring animal welfare benefits.

## Conclusions

We estimated that the annualised benefits of eradicating BVDV from Ireland exceeded the costs by a factor of 5 in the suckler beef sector and by a factor of 14 in the dairy sector. This is an important prerequisite for a successful eradication campaign.

## Competing interests

The authors declare that they have no competing interests.

## Authors’ contributions

AWS designed and conducted the cost benefit analysis, developed the model of finisher herds in consultation with RWH, updated and adapted the other models for this study and drafted the paper. RWH originally constructed the dairy model under the guidance of GJG & AWS, ran the selected simulations for the dairy model and summarised the results. GJG negotiated the research contract for SAC with AHI and managed the contract. He also provided specialist advice to the other authors on various aspects of the work. IH generated descriptive data for suckler, dairy and beef fattening herds; TH generated economic data; JOF and DG conceived of the study and participated in its design and coordination and helped to draft the manuscript. All authors read and approved the final manuscript.

## References

[B1] Gunn GJ Saatkamp HW Humphry RW Stott AW Assessing economic and social pressure for the control of bovine viral diarrhoea virus Prev Vet Med 2005 71 149 162 16243404 10.1016/j.prevetmed.2005.08.012

[B2] Houe H Epidemiological features and economical importance of bovine virus diarrhoea virus (BVDV) infections Vet Microbiol 1999 64 89 107 10.1016/S0378-1135(98)00262-4 10028165

[B3] Charleston B Fray M Baigent S Carr BV Morrison WI Establishment of persistent infection with non-cytopathic bovine viral diarrhoea virus in cattle is associated with a failure to induce type 1 interferon J Gen Virol 2001 82 1893 1897 11457995 10.1099/0022-1317-82-8-1893

[B4] Meadows D A study to investigate the use and application of BVDV vaccine in UK cattle Cattle Practice 2010 18 202 215

[B5] Heffernan C Nielsen L Thomson K Gunn GJ An exploration of the drivers to bio-security collective action among a sample of UK cattle and sheep farmers Prev Vet Med 2008 87 358 372 10.1016/j.prevetmed.2008.05.007 18692923

[B6] Lindberg A Brownlie J Gunn GJ Houe H Moennig V Saatkamp HW Sandvik T Valle PS The control of bovine viral diarrhoea virus in Europe: today and in the future Revue Scientifique et Technique-Office International des Epizooties 2006 25 961 979 17361763

[B7] Presi P Struchen R Knight-Jones T Scholl S Heim D Bovine viral diarrhea (BVD) eradication in Switzerland-Experiences of the first two years Prev Vet Med 2011 99 112 121 10.1016/j.prevetmed.2011.01.012 21371766

[B8] Heffernan C Misturelli F Nielsen L Gunn GJ Yu J Analysis of Pan-European attitudes to the eradication and control of bovine viral diarrhoea Vet Rec 2009 164 163 167 10.1136/vr.164.6.163 19202168

[B9] More SJ McKenzie K O'Flaherty J Doherty ML Cromie AR Magan MJ Setting priorities for non-regulatory animal health in Ireland: Results from an expert Policy Delphi study and a farmer priority identification survey Prev Vet Med 2010 95 198 207 10.1016/j.prevetmed.2010.04.011 20554068

[B10] Barrett DJ More SJ Graham DA O’Flaherty J Doherty ML Gunn HM Considerations on BVD eradication for the Irish livestock industry Irish veterinary Journal 2011 64 12 10.1186/2046-0481-64-12 21967764 PMC3199273

[B11] Gunn GJ Stott AW Humphry RW Modelling and costing BVD outbreaks in beef herds Vet J 2004 167 143 149 10.1016/S1090-0233(03)00112-6 14975388

[B12] Connolly L Kinsella A Quinlan G Moran B Teagasc, Rural Economy Research Centre 2009 National Farm Survey, Athenry, Ireland

[B13] Microsoft Microsoft Office Excel 2007 Part of Microsoft Office Professional Plus, Washington, USA

[B14] Stott AW Veerkamp RF Wassell TR The economics of fertility in the dairy herd Animal Science 1999 68 49 57

[B15] Stott AW Humphry RW Gunn GJ Modelling the effects of previous infection and re-infection on the costs of bovine viral diarrhoea outbreaks in beef herds Vet J 2010 185 138 143 10.1016/j.tvjl.2009.05.020 19709915

[B16] Stott AW Lloyd J Humphry RW Gunn GJ A linear programming approach to estimate the economic impact of bovine viral diarrhoea (BVD) at the whole-farm level in Scotland Prev Vet Med 2003 59 51 66 10.1016/S0167-5877(03)00062-X 12719017

[B17] Cowley DJB Aspects of Bovine Herpesvirus 1 and Bovine Virus Diarrhoea Virus infection in beef and dairy herds in the Republic of Ireland 2010 University College Dublin, MVM Thesis

[B18] Stott AW Gunn GJ Use of a benefit function to assess the relative investment potential of alternative farm animal disease prevention strategies Prev Vet Med 2008 84 179 193 10.1016/j.prevetmed.2007.12.001 18243379

[B19] Weldegebriel HT Gunn GJ Stott AW Evaluation of producer and consumer benefits resulting from eradication of bovine viral diarrhoea (BVD) in Scotland, United Kingdom Prev Vet Med 2009 88 49 56 10.1016/j.prevetmed.2008.07.001 18937987

[B20] Graham DA German A McLaren IE Fitzpatrick DA Testing of bulk tank milk from Northern Ireland dairy herds for viral RNA and antibody to bovine viral diarrhoea virus Vet Rec 2001 149 261 265 10.1136/vr.149.9.261 11558660

[B21] Ganser AG Brulisauer F McCormick BJJ Gunn GJ Stott AW Using enterprise gross output as an argument for endemic disease control at farm level 2010 201 212

[B22] Houben EHP Huirne RBM Dijkhuizen AA Optimal replacement of mastitic cows determined by a hierarchic markov process J Dairy Sci 1994 77 2975 2993 10.3168/jds.S0022-0302(94)77239-8 7836585

[B23] Valle PS Skjerve E Martin SW Larssen RB Ostera O Nyberg O Ten years of bovine virus diarrhoea virus (BVDV) control in Norway: a cost-benefit analysis Prev Vet Med 2005 72 189 207 10.1016/j.prevetmed.2005.07.017 16213612

[B24] Scottish Government a Bovine Viral Diarrhoea (BVD) An eradication scheme for Scotland – Consultation Paper, Scotland http://www.scotland.gov.uk/Publications/2010/06/29143957/0

[B25] Scottish Government b An analysis of the effects of BVD eradication in Scotland, Scotland http://www.scotland.gov.uk/Resource/Doc/915/0099584.doc

[B26] Gill M Smith P Wilkinson JM Mitigating climate change: the role of domestic livestock Animal 2010 4 323 333 10.1017/S1751731109004662 22443938

